# Positive and negative affect facilitate creativity motivation: Findings on the effects of habitual mood and experimentally induced emotion

**DOI:** 10.3389/fpsyg.2023.1014612

**Published:** 2023-01-26

**Authors:** Wu-jing He

**Affiliations:** Department of Special Education and Counselling, The Education University of Hong Kong, Hong Kong SAR, China

**Keywords:** creativity, motivation, affect, mood, emotion, experimental emotion induction

## Abstract

This research involved two investigations that examined the effects of two types of affect (i.e., mood and emotion) on creativity motivation. Study 1 examined the degree to which noninduced habitual mood impacted creativity motivation in the context of a group of junior secondary school students in Hong Kong (*n* = 588), while Study 2 examined the effect of the experimental manipulation of emotion induction on creativity motivation in the context of a group of undergraduate students in Hong Kong (*n* = 653). The Chinese version of the Creativity Motivation Scale, the International Positive and Negative Affect Schedule-Short Form, and the Affect Grid were employed to assess creativity motivation, mood, and emotional states, respectively. Interesting findings were obtained. First, both studies consistently demonstrated a facilitating role of positive and negative affect in creativity motivation. Second, both studies consistently showed that the impact of positive affect on creativity motivation was stronger than that of negative affect. While previous affect-creativity research has focused predominantly on the role of affect in the cognitive components of creativity and yielded mixed results, this research adds to the literature by showing that students’ motivation to engage in creativity-related behaviors can be influenced by a broad spectrum of affective experiences (i.e., positive and negative affect, stable and enduring moods, and momentary and mutable emotions). The theoretical and educational implications of the findings are highlighted.

## Introduction

Researchers are interested in exploring the influence of affect on human functioning, including creativity (Kühnel et al., 2022). There is general agreement that creative functioning is affected by affective states, although whether positive or negative affect facilitates or inhibits creative performance is still an ongoing debate (Chi and Lam, 2022). Interestingly, while creativity is commonly accepted as a multicomponent construct (Ivancovsky et al., 2021), creative performance has generally been assessed with divergent thinking and idea generation tasks, association tasks, creative problem-solving tasks, and general creativity performance measures in the existing affect–creativity literature, which focuses primarily on the cognitive components of creativity (Baas et al., 2008; Davis, 2009; Zielińska et al., 2022). The present study aimed to extend this line of research by examining the impact of affect on an alternative creativity component, namely, creativity motivation, which focuses on the driving force of creativity-related behaviors (Zhang et al., 2018).

### Research on the affect–creativity link

In the extensive research work regarding the affect–creativity link, affect is usually conceptualized as a broad term, referring to a subjective feeling state that incorporates both moods and emotions (e.g., Baas et al., 2008; Davis, 2009; He and Wong, 2022). Specifically, moods denote habitual, stable and long-lasting affective states, which are mild feelings that are experienced as diffuse psychological states. This type of affective state is weakly connected to specific stimuli or causal factors and lasts for 1 day or a few days/weeks; for example, experiencing a general, enduring, and diffuse psychological state of depression over 1 week, for which no direct or specific causal factors can be identified. Emotions, in contrast, represent the other type of affective experiences, which are volatile, momentary (lasting for seconds or minutes), and intense reactions in response to specific stimuli or causal factors such as a person, an object, or an event; for example, experiencing a notable change in one’s emotional state regarding the level of happiness in response to a musical stimulus such as Mozart’s Sonata (see also Fox and Moore, 2021). As He and Wong (2022) highlighted, “… mood and emotion are similar to each other in the sense that they are both subtypes of affect. However, they are different from each other in the sense that mood is a more stable and longer lasting affective state but less specific, less intense, and less likely to be triggered by a particular stimulus or event in the environment…” (p. 2). Moreover, following core affect theory (Russell, 2003) and valence-arousal theory (Baas et al., 2008), affect can be conceptualized by reference to two dimensions: arousal (activated or deactivated) and valence (positive or negative). Specifically, arousal refers to one’s readiness to attend to stimuli and engage in tasks, while valence refers to the intrinsic attractiveness of an affect, in which context positive-valence affect is intrinsically attractive while negative-valence affect is intrinsically aversive (Sambrano et al., 2021; Shin et al., 2022).

Much of the existing research on the affect–creativity link has focused primarily on the cognitive processes and thinking skills underlying creativity, in which context creative performance has been predominantly assessed with divergent thinking and idea generation tasks, association tasks, creative problem-solving tasks, and general creativity performance measures (Baas et al., 2008; Davis, 2009; Zielińska et al., 2022). Such research generally supports the beneficial role of positive affect. For instance, positive affect has been shown to facilitate divergent thinking (Mastria et al., 2021; Kühnel et al., 2022), broadened attentional scope (Paul et al., 2021), creative problem solving (Kumar et al., 2022), associative thinking (Frith et al., 2022), cognitive persistence in creativity tasks (Baas et al., 2011), and creative cognition (Eskine et al., 2020). With respect to the role of negative affect, however, research findings are equivocal. While some studies have reported that negative affect plays a facilitating role in creative thinking, divergent thinking, and creative problem solving (Zhan et al., 2020; Du et al., 2021), other studies have suggested that there are no such effects (Harada, 2021) or even that there is a negative effect (Acar et al., 2021; Mao et al., 2021). These inconsistent findings suggest that further empirical scrutiny is warranted to verify the role of affect in creativity.

### Extending affect–creativity research to creativity motivation

Creativity is understood to be a multicomponent construct (Ivancovsky et al., 2021) that relies not only on cognitive processes or thinking skills to combine existing knowledge in a novel and useful way but also on the motivation to transform existing knowledge into creative output by employing effective cognitive and thinking strategies (Bornstein, 2022). Motivation has been highlighted as an important engine that drives people to engage in a creative task and to sustain creative endeavors in many creativity theories, such as the componential theory of creativity (Amabile, 1996/2019), the investment theory of creativity (Sternberg, 2006), the systems model of creativity (Csikszentmihalyi, 2015), and the two-tiered componential model of creative thinking (Runco and Chand, 1995). According to these theoretical models, task motivation (especially intrinsic motivation) represents one of the critical components that works together with many other creativity components (e.g., cognitive components) to facilitate or inhibit creative performance. In contrast to cognitive components that concern psychological attributes in relation to cognitive processes, thinking skills, problem-solving strategies, and acquired knowledge, motivational components focus primarily on psychological attributes related to achievement goals, self-regulatory processes, and experience quality, which may increase or decrease one’s willingness to invest time and effort in creative endeavors (Baas et al., 2011; Darfler and Kalantari, 2022).

Taking the theoretical position that highlights the role of motivation in creativity, Zhang et al. (2018) recently proposed creativity motivation theory and introduced a new motivation construct (i.e., creativity motivation) to denote the force that drives an individual toward creativity-related behaviors. The theory further extends previous research by taking a two-dimensional approach to define creativity motivation, which consists of (a) the behavior disposition dimension and (b) the force dimension. While the former refers to the three forms of creativity-related behaviors (i.e., learning, doing, and accomplishing new things), the latter refers to the three forms of driving force that energize creativity-related behaviors, including (1) high-quality experiences (i.e., intrinsic motivation), (2) instrumental purpose (i.e., extrinsic motivation), and (3) value (i.e., the perceived importance of the creativity behavior in question). Creativity motivation theory offers a new insight to highlight that a more accurate and comprehensive understanding of creativity motivation can be achieved based on a 3 (forms of creativity-related behaviors) by 3 (forms of driving force) combination of the two dimensions in defining the construct (see also Li et al., 2021).

Although the role of motivation in creativity has been well recognized, surprisingly little empirical research has been conducted to examine the impact of affect on creativity motivation, especially compared to the large amount of research that has focused on the role of affect in the cognitive processes and thinking skills underlying creativity. Empirical questions pertaining to the role of positive and negative affect in creativity motivation remain under researched. The present study aimed to fill this research gap. Several theories shed light on the expected relationship between affect and motivation in general, although a specific theory focusing on the role of affect in creativity motivation remains lacking. On the one hand, some theories emphasize the beneficial role of positive affect in motivation. For example, affect-as-information theory (Schwarz, 1990) postulates that people with a positive mood are more likely to use heuristics to interpret their affective experiences, which tends to prime favorable judgments and to result in enhanced motivation to engage in the target tasks. This theory suggests that the safety signal elicited by positive affective states enhances individuals’ motives to seek stimulation and pursue incentives (Zanger et al., 2022). Similarly, the broaden-and-build theory (Fredrickson, 2001) holds that people who exhibit a positive emotional state tend to experience stronger urges to act in a greater variety of ways (Stanley and Schutte, 2023).

On the other hand, there are also theories that highlight the beneficial role of negative affect in motivation. For example, control theory (Martin and Stoner, 1996) suggests that negative emotion may signal to individuals who their current progress faces certain expectations, thereby driving them to invest more motivated effort to reach their expected goals. Another theory that emphasizes motivational force as part of a mood-repair strategy suggests that people with a sad mood tend to employ effortful and motivated processing to complete tasks as a means of distracting themselves from negative thoughts (Erber and Erber, 1994). Moreover, the hedonistic discounting hypothesis (Forgas, 2013) postulates that people in a negative mood may place greater value on the expected hedonistic benefits of success and have a stronger motive to invest more effort in tasks than people in a positive mood (see also Madrid and Patterson, 2018, 2021).

In summary, all of these theories postulate the influential role of affect in task motivation. While some theories highlight the facilitating role of positive affect and others emphasize the beneficial role of negative affect on task motivation, this fact does not necessarily imply that the effects of positive and negative affect on task motivation are mutually exclusive. Indeed, research findings have shown that positive moods are linked to enhanced motivation to become involved in tasks that are perceived as fun and intrinsically rewarding and to participate in situations in which enjoyment is highlighted; in contrast, negative moods are linked to increased effort investment in tasks that are perceived as serious and extrinsically rewarding and increased involvement in situations in which meeting performance standards are emphasized (Baas et al., 2011; Hwang and Choi, 2020; Zielińska et al., 2022). These findings imply that both positive and negative affect can enhance task motivation through different routes or mechanisms.

Drawing on these theoretical perspectives, it is expected that both positive and negative affect can contribute to creativity motivation – a specific type of motivation driving creativity-related behaviors (Zhang et al., 2018). The objective of this study was thus to empirically examine the expected facilitating role of both positive and negative affect in creativity motivation. While affect has been understood as an umbrella term referring to a subjective feeling state that incorporates both mood and emotional state, which share similar and different characteristics in terms of intensity, frequency, duration, and specificity, the overarching research question of the study was to understand whether the two types of affect (i.e., mood and emotional state) in both positive and negative valence could facilitate creativity motivation. Specifically, two investigations were carried out to address this research question. While the first investigation (Study 1) focused on the role of positive and negative moods in creativity motivation, the second investigation (Study 2) focused on the role of positive and negative emotions in creativity motivation. Because mood is understood as a habitual, stable and long-lasting affect that is weakly connected to specific stimuli or causal factors (Fox and Moore, 2021; He and Wong, 2022), Study 1 involved a correlational study to uncover the degree to which positive and negative habitual mood could explain individuals’ variance in creativity motivation. In contrast, because emotion is understood as a volatile, momentary, and intense affective reaction in response to specific stimuli or causal factors (Fox and Moore, 2021; He and Wong, 2022), Study 2 involved an experimental study to determine whether the experimental manipulation of emotion induction in positive or negative valence by using musical stimuli could change creativity motivation. By collecting data from both a correlational and an experimental study using different methodologies to examine two different types of affective experiences, it was expected that a more comprehensive understanding could be achieved with respect to the roles of affect in creativity motivation.

## Study 1

### Research question and hypotheses

The research question of Study 1 was to uncover to what degree positive and negative moods impact creativity motivation. While mood represents a subtype of affective experience that is stable, enduring, less intense, and weakly connected to specific stimuli or causal factors, this study did not include affective induction *via* specific affect-evoking stimuli and focused instead on noninduced habitual mood.

Drawing upon the affect-as-information and broaden-and-build theories, the following hypothesis was formulated:

***Hypothesis 1a (H1a).*** Positive mood is positively predictive of creativity motivation.

Drawing upon control theory, mood-repair theory, and the hedonistic discounting hypothesis, the following hypothesis was formulated:

***Hypothesis 1b (H1b).*** Negative mood is positively predictive of creativity motivation.

### Methods

#### Participants

A convenience sampling procedure was followed in the present study. Participants were recruited from six coeducational secondary schools in various districts of Hong Kong. All six schools were government-aided schools and admitted students from diverse backgrounds; however, most students were from middle-class or lower-middle-class socioeconomic backgrounds. After obtaining permission to recruit participants from the school principals, the researchers distributed the information package to students and instructed them to give it to their parents. While it was assured that participation was completely voluntary, participation was restricted to those students who returned written parental informed consent and child assent forms. Participants were also excluded if they (a) indicated difficulties in reading and comprehending Chinese, (b) indicated any mood problems in the last 6 months, or (c) indicated any treatment for mental health problems in the last 6 months. Eight participants (1.34%) were excluded from the analyses due to missing data in the instruments used in the study. The final sample consisted of 588 junior secondary school students (49.3% female) in grades 7 through 9 and between the ages of 13 and 17 years (*M* = 15.1, *SD* = 1.24). All participants were ethnically Chinese.

#### Instruments

##### Moods

To assess the habitual mood of individuals in this sample, the Chinese version of the short form of the International Positive and Negative Affect Schedule (I-PANAS-SF; Liu et al., 2020) was employed. The I-PANAS-SF consists of 10 items, which include five items measuring positive affect (PA, i.e., active, alert, attentive, determined, inspired) and five items measuring negative affect (NA, i.e., afraid, ashamed, hostile, nervous, upset). Participants respond on a 5-point Likert-type scale with answers ranging from 1 (never) to 5 (always) to indicate how often they have experienced the feelings described by each item over the past few weeks. The I-PANAS-SF has been widely used by previous studies involving student samples and has demonstrated good validity and reliability (e.g., Earl et al., 2019), in which context the convergent and criterion-related validities of the instrument were supported by its significant correlations with the original and full versions of the PANAS. And the construct validity of the two-factor model was supported by the results of confirmatory factor analysis (CFA), with CFI = 0.94 and RMSEA = 0.066 (see Thompson, 2007; Liu et al., 2020).

In the present study, the obtained fit indices of a CFA also lent support to the two-factor model of the scale (*χ*^2^ = 97.8, df = 34, *χ*^2^/df = 2.88, CFI = 0.951, TLI = 0.950, RMSEA = 0.057, SRMR = 0.061), confirming the construct validity of the scale in this Chinese student sample. Furthermore, the convergent validity of the scale was assessed using the average variance extracted (AVE) and the composite reliability (CR), while the discriminant validity was assessed using the HeteroTrait-MonoTrait ratio of correlations (HTMT). The results revealed that an AVE value greater than 0.50 was obtained for both PA (AVE = 0.62) and NA (AVE = 0.57) while a CR value greater than 0.70 was achieved for both PA (AVE = 0.89) and NA (CR = 0.87), confirming the convergent validity of the scale. Moreover, the obtained HTMT value (i.e., 0.19) was smaller than 0.90, lending support to the discriminant validity of the scale (Hair et al., 2021). With respect to reliability, *Cronbach’s α* (*α* = 0.81 for PA; *α* = 0.80 for NA) and *McDonald’s ω* coefficients (*ω* = 0.83 for PA; *ω* = 0.81 for NA) greater than 0.70 were obtained, confirming an adequate internal consistency (Hayes and Coutts, 2020).

##### Creativity motivation

To assess creativity motivation, the Creativity Motivation Scale (CMS; Zhang et al., 2018) was adapted and translated into Chinese *via* a back-translation procedure. The CMS is a 9-item self-report questionnaire developed based on creativity motivation theory (Zhang et al., 2018), which conceptualizes creativity motivation as the force that drives individuals to engage in creative activities that are characterized as learning, doing, and accomplishing new things. Specifically, learning new things refers to one’s discovery of useful knowledge and skills related to creativity. Doing new things pertains to the behaviors involved in incorporating existing ideas, knowledge, and skills into actions. Finally, accomplishing new things indicates the behaviors involved in bringing a perceptible product into being in a completed state. The three types of motivational forces specified in creativity motivation theory include (1) high-quality experience (i.e., an intrinsic motivation that refers to the sheer pleasure and enjoyment experienced while performing a creativity activity), (2) instrumental purpose (i.e., an extrinsic motivation that pertains to purposes outside of the task itself and consideration of external rewards and utility while engaging in a creativity activity), and (3) value (i.e., the level of importance that individuals ascribe to creativity and desirability). See also Hennessey (2015) and Tierney (2015).

Each item of the CMS was developed by combining one type of motivational force and one type of creativity-related behavior. A sample item highlighting a combination of high-quality experience and doing new things is as follows: “I experience pleasure when I do something in my own original way.” A sample item emphasizing a combination of instrumental purpose and learning new things is as follows: “It is useful to discover new things that I have never seen before.” A sample item focusing on a combination of value and accomplishing new things is as follows: “It is important to bring a perceptible product to completion.” Participants responded on a 6-point Likert-type scale with answers ranging from 1 (strongly disagree) to 6 (strongly agree) to indicate the extent to which they agreed or disagreed with these statements. Supporting evidence for the construct validity of the CMS was found in culturally diverse samples from multiple countries, such as Chile, China, Kosovo, Russia, Saudi Arabia, and Turkey, which confirmed either a three-factor model (i.e., learn, do, and accomplish new things) with acceptable goodness-of-fit indices (i.e., CFI = 0.94–0.97, RMSEA = 0.051–0.075) and a one-factor model (i.e., using CMS as a total score to indicate the level of creativity motivation; CFI = 0.92–0.97, RMSEA = 0.052–0.090; see Zhang et al., 2018). For the current sample of Chinese students in Hong Kong, the results of CFA showed that the one-factor model had good fit indices (*χ*^2^ = 75.1, df = 27, *χ*^2^/df = 2.78, CFI = 0.964, TLI = 0.958, RMSEA = 0.055, SRMR = 0.042). Furthermore, the calculated AVE (i.e., 0.51) and CR (i.e., 0.95) were greater than 0.50 and 0.70, respectively, confirming the convergent validity of the scale. In addition, the reliability of the scale was supported by the high *Cronbach’s α* (*α* = 0.83) and *McDonald’s ω* coefficients (*ω* = 0.83).

#### Procedure

The instruments were administered collectively to classroom groups of 20 to 30 students in a regular classroom setting. The participants first completed the I-PANAS-SF and then completed the CMS according to the standard instructions.

#### Data analysis

SPSS software (version 26.0) was used for statistical analysis. First, descriptive analysis was performed, and the normal distribution of variables was confirmed with the Kolmogorov–Smirnov test. Second, to test H1a and H1b, which contend that positive (H1a) and negative mood (H1b) are positively predictive of creativity motivation, multiple regression analyses were performed subsequent to a Pearson correlation analysis. Pearson correlation analysis was performed to reveal the association between demographic variables (i.e., gender, age, education level, parents’ education level), positive and negative mood (i.e., PA and NA scores), and creativity motivation (i.e., CMS score). Multiple regression analyses were carried out to examine the influence of predictor variables on an outcome variable. Specifically, hierarchical multiple regression analyses were performed, in which context creativity motivation was established as the criterion, while PA and NA scores were identified as the predictors. Demographic variables that showed significant correlations with creativity motivation were included in Step 1 to control for their possible covariate effects on creativity motivation. The predictor variables (i.e., PA and NA scores) were then included in Step 2 to test for their unique contribution to creativity motivation. A *p* of less than 0.05 was regarded as a statistically significant value in all cases. The adjusted *R*^2^ statistics were used to evaluate the goodness of fit of the model, while the *B* and *β* statistics were used to determine the degree to which each predictor variable could influence the outcome variable. Cohen’s ƒ^2^ (Cohen, 2013) was used to calculate the effect size of the predictor variables within the multiple regression model. According to guidelines of Cohen (2013), the criteria to interpret the magnitude of effect size were as follows: small (ƒ^2^ ≥ 0.02), medium (ƒ^2^ ≥ 0.15), or large (ƒ^2^ ≥ 0.35).

### Results

Table 1 presents the descriptive statistics of and the correlation matrix among PA, NA, creativity motivation and demographic variables, while Table 2 displays the results of hierarchical regression analyses. As expected, the Pearson correlation coefficient statistics indicated that creativity motivation was positively correlated with PA (*r* = 0.312; *p* = 0.001) and NA (*r* = 0.190; *p* = 0.012). Furthermore, significant correlations were found for creativity motivation for the demographic variables, including gender (*r* = 0.126; *p* = 0.024) and parents’ education level (*r* = 0.147–0.185; *p* = 0.016–0.020). However, no significant correlations were found for demographic variables such as age (*r* = 0.011; *p* = 0.58) and participants’ education level (*r* = 0.012; *p* = 0.55).

**Table 1:** Means, standard deviations (SDs), and correlations between variables of Study 1 (*n* = 588).

	1	2	3	4	5	6	7	8
1. Gender^a^	1.00							
2. Age	0.021	1.00						
3. Education	0.009	0.912^***^	1.00					
4. Mather’ education	0.001	0.007	0.009	1.00				
5. Father’s education	0.002	0.005	0.008	0.561^***^	1.00			
6. PA	0.128^*^	0.036	0.010	0.164^**^	0.122^*^	1.00		
7. NA	−0.125^*^	0.009	0.011	0.091	−0.095	−0.098	1.00	
8. Creativity motivation	0.126^*^	0.011	0.012	0.147^*^	0.185^**^	0.312^***^	0.190^**^	1.00
Mean	–	15.2	8.04	13.3	14.1	3.21	2.99	3.87
SD	–	1.06	1.97	2.88	3.12	0.68	0.74	0.91

a 1 = M, 0 = F; PA, Positive affect; NA, Negative affect.

* *p* < 0.05.

** *p* < 0.01.

*** *p* < 0.001.

**Table 2:** Results of hierarchical regression analyses predicting creativity motivation in study 1 (*n* = 588).

	Predicting creativity motivation						
	*B*	SE	*β*	95% CI	*t*	*p*	*ƒ* ^2^
**Step 1**							
Gender^a^	0.13	0.09	0.07	[−0.03, 0.13]	0.63	0.860	0.000
Mother’s education	0.08	0.04	0.13	[0.04, 0.09]	4.71	0.009	0.016
Father’s education	0.07	0.04	0.12	[0.03, 0.08]	2.06	0.011	0.012
*R* ^2^_adj._	–	–	–	–	0.070	0.003	–
**Step 2**							
PA	0.20	0.03	0.22	[0.16, 0.20]	6.31	0.001	0.182
NA	0.10	0.04	0.13	[0.07, 0.12]	5.01	0.008	0.131
*R* ^2^_adj._	–	–	–	–	0.165	0.004	–
∆*R*^2^_adj._	–	–	–	–	0.095	0.001	–

a 1 = Male, 0 = Female

As shown in Table 2, the results of hierarchical regression analyses revealed that PA and NA together accounted for 9.5% of the variance in creativity motivation at a statistically significant level (∆*R*^2^_adj._ = 0.095, ∆*F*(4, 583) = 33.90, *p* = 0.001) after controlling for the effects of demographic variables (*R*^2^_adj._ = 0.070, *F*(2, 585) = 9.91, *p* = 0.003), in which context both PA and NA predicted creativity motivation in a positive direction. Specifically, the *B* statistics (i.e., *B* = 0.20) revealed that creativity motivation showed an increase of 0.20 points in the CMS for each one-point increase on the PA scale. With respect to the effect of NA, the *B* statistics (i.e., *B* = 0.10) revealed that creativity motivation showed an increase of 0.10 points in the CMS for each one-point increase on the NA scale. Furthermore, the *β* statistics suggest that PA (*β* = 0.22) showed stronger predictive power than NA (*β* = 0.13) in accounting for the variance in creativity motivation. Overall, these results support the hypotheses with respect to the facilitating role of positive (H1a) and negative mood (H1b) in creativity motivation. These results further illustrated that positive mood had a stronger predictive power than negative mood in predicting creativity motivation.

## Study 2

### Research question and hypotheses

The research question of Study 2 was to understand the role of another type of affect (i.e., emotion) in creativity motivation. As emotion represents a type of affective experience that is volatile, momentary, intense, and directed toward specific external stimuli or causal factors, this study was designed to examine whether the experimental manipulation of emotion induction in either positive or negative valence could change creativity motivation. Musical stimuli are used for emotion induction in this investigation because such stimuli have been shown to be effective with respect to inducing positive and negative emotions in previous research (Fox and Moore, 2021; Shin et al., 2022).

Drawing upon the affect-as-information and broaden-and-build theories, the following hypothesis was formulated:

***Hypothesis 2a (H2a).*** Experimental emotion induction in a positive valence enhances creativity motivation when compared with a control condition.

Drawing upon control theory, mood-repair theory, and the hedonistic discounting hypothesis, the following hypothesis was formulated:

***Hypothesis 2b (H2b).*** Experimental emotion induction in a negative valence enhances creativity motivation when compared with a control condition.

### Methods

#### Participants

A convenience sampling procedure was followed in the present study. Undergraduate students from three universities in Hong Kong were invited to take part in the study on a voluntary basis. Participation was restricted to those students who returned informed written consent after attending a debriefing session in which they were assured that participation in the study was completely voluntary, anonymous and confidential. Participants were also excluded if they (a) indicated difficulties in reading and comprehending Chinese, (b) indicated any mood problems in the last 6 months, (c) indicated any treatment for mental health problems in the last 6 months, or (d) indicated any hearing impairment in the last 6 months. Eleven participants (1.66%) were excluded from the analyses due to missing data in the instruments used in the study. The final sample consisted of 653 participants (51.6% female). All participants were ethnically Chinese, and their ages ranged from 19 to 26 years (*M* = 21.7; *SD* = 1.41). See Table 3 for the demographic characteristics of this sample.

**Table 3:** Demographic and mood characteristics of the sample in Study 2 (*n* = 653).

	Positive emotion group (*n* = 218)	Negative emotion group (*n* = 219)	Control group (*n* = 216)	*F*	*p*	*η_p_* ^2^
	Mean (±SD)	Mean (±SD)	Mean (±SD)			
Age	21.8 (±1.40)	21.7 (±1.54)	21.6 (±1.28)	2.24	0.111	0.003
Education	15.5 (±1.01)	15.6 (±1.32)	15.4 (±1.07)	0.95	0.390	0.001
GPA	3.00 (±0.21)	2.99 (±0.21)	3.02 (±0.16)	0.99	0.371	0.001
Mother’s education	13.2 (±1.05)	13.2 (±1.29)	13.4 (±1.08)	1.50	0.220	0.002
Father’s education	14.0 (±0.87)	14.1 (±1.09)	13.9 (±1.07)	1.91	0.154	0.003
**Mood**						
PA	3.13 (±1.17)	3.16 (±1.23)	3.15 (±1.24)	0.04	0.963	0.000
NA	2.91 (±1.22)	2.95 (±1.30)	2.81 (±1.11)	0.70	0.501	0.000
	**Frequency (%)**	**Frequency (%)**	**Frequency (%)**	***χ***^**2**^	***p***	**–**
**Gender**				0.03	0.961	–
Male	105 (48.2)	107 (48.9)	104 (48.1)			
Female	113 (51.8)	112 (51.1)	112 (51.9)			

#### Materials

##### Musical stimuli

The musical stimuli used in this study consisted of 10-min excerpts of music that had been shown by past studies (e.g., Husain et al., 2002; Schellenberg et al., 2007) to be able to induce positive (i.e., Mozart’s Sonata for Two Pianos in D Major) or negative emotions (i.e., Albinoni’s Adagio in G Minor). The applicability of these musical stimuli has been supported in a Chinese student sample in Hong Kong by a study that examined the effect of music listening on emotions and creative thinking (He et al., 2017).

##### Instruments

The adapted Chinese Affect Grid (Russell et al., 1989) was employed to assess the emotional changes caused by the musical stimuli, which was regarded as an effective measure for the rapid and repeated capture of fast changes in emotional states in response to stimuli such as music (Russell and Gobet, 2012; He et al., 2017). The convergent and criterion-related validities of the affect grid have been supported by previous studies that showed significant correlations between the Grid and other measures of affect, for example, the PANAS and the profile of mood states (Killgore, 1998). The Affect Grid measures emotions by reference to two dimensions: arousal-sleepiness (i.e., arousal) and pleasure-displeasure (i.e., valence). Accordingly, this measure consists of two scores based on these two dimensions: (1) the arousal score, which ranges from 1 (sleepiness) to 9 (high arousal), and (2) the valence score, which ranges from 1 (displeasure) to 9 (pleasure). See He et al. (2017, p. 5) for a visual presentation of the grid. Participants are instructed to place a single mark within the grid to indicate their emotional state. A higher score on the arousal dimension indicates a higher level of readiness to respond to stimuli, and a higher score on the valence dimension indicates a more positive valence with respect to the feeling of pleasure.

Similar to the method used in Study 1, the Chinese CMS was employed to assess creativity motivation. Because daily habitual mood was shown by Study 1 to correlate significantly with creativity motivation, the Chinese I-PANAS-SF was also used in Study 2 to assess the general mood of this sample with the aim of controlling for its covariate effect on creativity motivation. See the Methods section of Study 1 for details concerning the Chinese CMS and I-PANAS-SF. For this university student sample, supporting evidence for the reliability and validity of the I-PANAS-SF and CMS were obtained. For the I-PANAS-SF, the obtained fit indices supported the construct validity of the two-factor model (*χ*^2^ = 89.4, df = 34, *χ*^2^/df = 2.63, CFI = 0.963, RMSEA = 0.055). The convergent validity of the scale was supported by the AVE values ranging between 0.66 (for NA) and 0.70 (for PA), and the CR values ranging between 0.91 (for NA) and 0.92 (for PA). The discriminant validity of the scale was supported by the HTMT (i.e., 0.21). The results also showed evidence for a good internal consistency of the PA (*α* = 0.88; *ω* = 0.89) and NA subscales (*α* = 0.82; *ω* = 0.83). With respect to the CMS, good fit indices were obtained to support the construct validity of the one-factor model (*χ*^2^ = 79.8, df = 27, *χ*^2^/df = 2.96, CFI = 0.957, RMSEA = 0.051). The convergent validity was established with AVE = 0.51 and CR = 0.95. Good internal consistency of the scale was supported by the *Cronbach’s α =* 0.88 and *McDonald’s ω* = 0.86.

#### Procedure

Prior to emotion induction, participants completed the CMS and the Affect Grid as a pretest to assess their baseline level of creativity motivation and their emotional state, respectively. Participants’ habitual mood was also assessed *via* the I-PANAS-SF. Participants were assigned to three groups of “equivalent creativity motivation, emotional states, and habitual mood” based on their scores on the CMS, Affect Grid, and I-PANAS-SF, respectively. These three groups included (1) a positive emotion induction group (listening to Mozart; *n* = 218, 51.8% female), (2) a negative emotion induction group (listening to Albinoni; *n* = 219, 51.1% female), and (3) a control group (sitting in silence; *n* = 216, 51.9% female). See Table 3 for the mood characteristics of the sample and Table 4 for the summary statistics concerning creativity motivation and emotional state (i.e., participants’ arousal and valence scores). The three groups were matched in terms of their baseline creativity motivation, emotional state, and habitual mood (all *F*s[2, 650] ≤ 0.70, *p*s ≥ 0.50). Moreover, there were no significant differences among the three groups in terms of age, education, grade point average, parents’ education (all *F*s[2, 650] ≤ 2.24, *p*s ≥ 0.11) or gender distribution (*χ*^2^ = 0.03, *p* = 0.99).

**Table 4:** Pre-and post-test measures of emotional state and creativity motivation in Study 2 (*n* = 653).

	Positive emotion group (*n* = 218)	Negative emotion group (*n* = 219)	Control group (*n* = 216)	F	p	*η_p_* ^2^
	Mean (±SD)	Mean (±SD)	Mean (±SD)			
**Pre-test (T1)**						
Emotional state						
Arousal	4.62 (±1.13)	4.58 (±1.32)	4.60 (±1.13)	0.28	0.762	0.000
Valence	4.77 (±1.04)	4.85 (±1.33)	4.79 (±1.19)	0.25	0.784	0.000
Creativity motivation	3.78 (±0.97)	3.77 (±1.20)	3.79 (±0.76)	0.05	0.961	0.000
**Post-test (T2)**						
Emotional state						
Arousal	5.86 (±1.79)	5.33 (±1.71)	4.71 (±0.83)	27.0	0.003	0.120
Valence	6.10 (±1.90)	3.76 (±1.20)	4.81 (±0.99)	40.3	0.001	0.160
Creativity motivation	4.53 (±0.95)	4.14 (±1.24)	3.73 (±0.89)	32.7	0.002	0.140

In the emotion induction experiment, the two experimental groups were exposed to a stimulus condition for 10 min and then tested immediately thereafter by employing the affect grid and creativity motivation as a posttest. The positive and negative emotion induction groups were exposed to Mozart and Albinoni, respectively, for 10 min, while the control group was instructed to sit in silence for 10 min.

#### Data analysis

Statistical analyses were conducted with SPSS software (version 26.0). First, descriptive analysis was performed, and the normal distribution of variables was confirmed with the Kolmogorov–Smirnov test. Second, prior to hypothesis testing, an emotion manipulation check was performed to determine whether the expected emotions were successfully induced in the emotion induction experiment, in which context a 3 (groups: the positive emotion induction group vs. the negative emotion induction group vs. the control group) × 2 (time points: pretest vs. posttest) mixed design analysis of variance (ANOVA) was performed on the arousal and valence scores. Third, to test H2a and H2b, which contend that experimental manipulation of emotion induction in either a positive (H2a) or a negative valence (H2b) enhances creativity motivation, a mixed design of analysis of covariance (ANCOVA) was conducted on the creativity motivation score (i.e., the CMS score) for the three groups (the positive emotion induction group vs. the negative emotion induction group vs. the control group) that featured 2 time points (pre-vs. posttest) and controlled for the possible covariate effect of habitual mood. In the ANOVA and ANCOVA tests, significant interaction and main effects were followed up with Bonferroni-corrected host hoc t tests for subsequent group comparisons and simple t tests for pre-and posttest comparisons, respectively. A *p* of less than 0.05 was regarded as a statistically significant value in all cases. Effect sizes were calculated using partial eta squared (*η_p_*^2^). As recommended by Cohen (2013), the criteria to interpret the magnitude of effect size were as follows: small (*η_p_*^2^ ≥ 0.01), medium (*η_p_*^2^ ≥ 0.06), or large (*η_p_*^2^ ≥ 0.14).

### Results

#### Manipulation check

**Figure 1:**
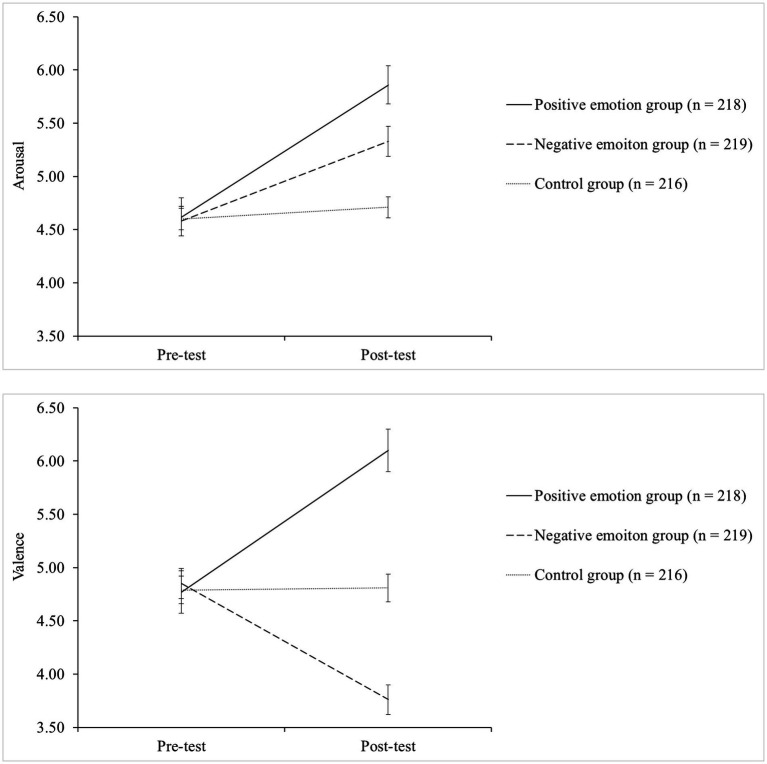
Changes in the arousal and valence scores between the pre-and post-test conditions for the three groups.

Figure 1 displays the changing patterns of the arousal and valence scores between the pre-and posttest conditions across the three groups. The results of a 3 (groups) × 2 (time points) mixed design ANOVA revealed a significant group × time interaction effect for both arousal (Wilks’ Lambda = 0.93, *F*[2, 650] = 27.2, *p* = 0.004, *η_p_*^2^ = 0.09) and valence (Wilks’ Lambda = 0.75, *F* [2, 650] = 109.16, *p* = 0.001, *η_p_*^2^ = 0.25), suggesting that the changes in the arousal and valence scores across the two time points were different across the three groups.

Hence, subsequent repeated-measures ANOVAs were performed separately for the three groups for both arousal and valence. The results confirmed that the emotion induction procedure was successful. In particular, the positive emotion induction group showed a statistically significant increase in valence, indicating an increased level of pleasure (Wilks’ Lambda = 0.68, *F*[1, 217] = 101.38, *p* = 0.001, *η_p_*^2^ = 0.32). In contrast, the negative emotion induction group demonstrated a statistically significant decrease in valence, illustrating a decreased level of pleasure or an increased level of displeasure (Wilks’ Lambda = 0.71, *F*[1, 218] = 88.29, *p* = 0.001, *η_p_*^2^ = 0.24). In the control group, no significant change was observed (Wilks’ Lambda = 1.00, *F*[1, 215] = 0.08, *p* = 0.77, *η_p_*^2^ = 0.00). With respect to the changes in arousal scores, a statistically significant increase was observed for both the positive (Wilks’ Lambda = 0.62, *F*[1, 217] = 135.38, *p* = 0.001, *η_p_*^2^ = 0.38) and negative (Wilks’ Lambda = 0.86, *F*[1, 218] = 35.06, *p* = 0.003, *η_p_*^2^ = 0.14) emotion induction groups. In the control group, as expected, no significant change was observed (Wilks’ Lambda = 0.99, *F*[1, 215] = 1.92, *p* = 0.17, *η_p_*^2^ = 0.01).

#### Hypothesis testing

**Figure 2:**
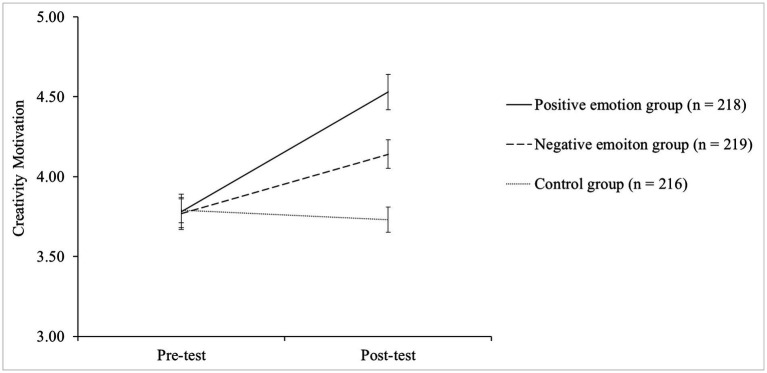
Changes in creativity motivation between the pre-and post-test conditions for the three groups.

Figure 2 presents the changes in creativity motivation as indicated by the CMS score between the pre-and posttest conditions across the three groups. The results of a 3 (groups) × 2 (time points) mixed design ANCOVA revealed a significant group × time interaction effect (Wilks’ Lambda = 0.58, *F*[2, 648] = 97.6, *p* = 0.002, *η_p_*^2^ = 0.48) controlled for the possible covariate effect of habitual mood. The significant interaction effect suggested that the changes in the CMS score across the two time points were different across the three groups. Hence, subsequent repeated-measures ANCOVAs were performed separately for the three groups to examine the changes in the CMS score from the pretest to posttest in the three conditions. As predicted, a statistically significant increase in the CMS score was observed in both the positive emotion induction group (Wilks’ Lambda = 0.70, *F*[1, 215] = 90.4, *p* = 0.001, *η_p_*^2^ = 0.24) and the negative emotion induction group (Wilks’ Lambda = 0.98, *F*[1, 216] = 9.21, *p* = 0.048, *η_p_*^2^ = 0.08), thus suggesting that either positive or negative emotion induction increases creativity motivation. These results confirmed H2a and H2b. Furthermore, the obtained effect sizes revealed that positive emotion induction (*η_p_*^2^ = 0.24) demonstrated a stronger effect than negative emotion induction (*η_p_*^2^ = 0.08) in regard to increasing creativity motivation. With respect to the control group, as expected, no significant change was observed (Wilks’ Lambda = 1.00, *F*[1, 213] = 0.03, *p* = 0.86, *η_p_*^2^ = 0.00).

The results revealed statistically significant differences across the three groups (*F* [2, 648] = 77.8, *p* = 0.001, *η_p_*^2^ = 0.41), suggesting that the three groups showed significantly different degrees of changes in creativity motivation between the pre-and posttest conditions. Subsequent analyses of *post hoc* pairwise comparisons employing the Bonferroni procedure to adjust for multiple comparisons illustrated that the positive emotion induction group showed a significantly higher degree of change in the CMS score (∆CMS score = 0.75) than the negative emotion induction group (∆CMS score = 0.37; *t* = 0.20, *p* = 0.000) and the control group (∆CMS score = −0.06; *t* = −0.80, *p* = 0.000). Moreover, the negative music group (∆CMS score = 0.37) also showed a significantly higher degree of change in the CMS score than the control group (∆CMS score = −0.06; *t* = −0.60, *p* = 0.000). These results again support H2a and H2b by showing that both positively and negatively induced emotional states can facilitate creativity motivation. The results further revealed that positively induced emotional states had a stronger impact in this context than did negatively induced emotional states.

## Discussion

It has been argued that affect informs motivational function (Kim et al., 2022). While some theories (e.g., affect-as-information and broaden-and-build theories) postulate a facilitating role of positive affect in task motivation, other theories (e.g., control theory, mood-repair theory, and the hedonistic discounting hypothesis) contend for a beneficial effect of negative affect on task motivation (see Madrid and Patterson, 2018, 2021). These theoretical positions were supported in the present study based on two empirical investigations that examined the effect of two types of affective experience on a new motivation construct (i.e., creativity motivation; Zhang et al., 2018). In the first investigation (Study 1), evidence was found to support the significant impact of noninduced habitual mood on creativity motivation in a group of junior secondary school students. Both positive and negative mood were found to be positively predictive of creativity motivation, confirming H1a and H1b. The results of effect sizes also revealed that positive mood was a stronger predictor than negative mood of creativity motivation. In the second investigation (Study 2), further analysis was performed to generalize the findings of Study 1 by extending the study in three ways: (1) extending the examination of the effect of noninduced habitual mood to exploring the effect of the experimental manipulation of emotion induction; (2) extending the use of an adolescent sample of secondary school students to the use of an emerging adult sample of university students; and (3) extending the use of a correlational design to applying an experimental design using standard emotion induction procedures. Similar to the findings obtained in Study 1, the results of Study 2 illustrated that the experimental manipulation of emotion induction in both a positive and a negative valence was effective in enhancing creativity motivation, confirming H2a and H2b. The results of effect sizes further illustrated that positive emotional induction showed a stronger effect than negative emotional induction in this context.

Taken together, the findings obtained in Studies 1 and 2 consistently demonstrated a facilitating role of both positive and negative affect in creativity motivation. These findings, on the one hand, add to the literature with respect to the motivational benefits of positive affect (e.g., Zanger et al., 2022; Stanley and Schutte, 2023) and lend empirical support to affect-as-information theory (Schwarz, 1990) and broaden-and-build theory (Fredrickson, 2001). On the other hand, these findings also add to the literature with respect to the motivational benefits of negative affect (e.g., Park et al., 2019; Zheng et al., 2022) and lend empirical support to control theory (Martin and Stoner, 1996), the account of mood as a repair strategy (Erber and Erber, 1994), and the hedonistic discounting hypothesis (Forgas, 2013). These findings enrich the existing affect-motivation literature by showing that one’s motivation to engage in creativity-related behaviors can be influenced by a broad spectrum of affective experiences, including a trait-like (i.e., mood) and a state-like affect (i.e., emotional state) in either a positive or a negative valence. These findings also extend the affect-motivation research by showing that the beneficial effect of both positive and negative affect on creativity motivation can be generalized across samples of different age groups ranging from adolescents to emerging adults. Furthermore, the experimental design in Study 2 allows for the interpretation of a possible cause-and-effect relationship between affective states and creativity motivation.

The finding concerning the facilitating role of both positive and negative affect in creativity motivation may be related to the nature of positive and negative affect, which has been understood as two separate and orthogonal affective dimensions but not ends of a single bipolar continuum (Lan et al., 2022). On the one hand, the independence of these affective dimensions makes it possible that feeling positively or feeling negatively may involve different psychological mechanisms (Jankowiak et al., 2022). On the other hand, however, that independence can also make it possible that feeling positively or negatively may involve similar and parallel experiences leading to similar outcomes, such as the enhanced creativity motivation highlighted in the present research. Specifically, people may enhance their motivation to engage in a creativity task because of the favorable judgments and the strong urges they experience in a positive affective state (Zanger et al., 2022; Stanley and Schutte, 2023). In parallel, people may also enhance their motivation to engage in a creativity task because of the feeling of inadequacies and threat in a negative affective state, which drives them to put more effort into engaging in the creativity task to meet certain expectations. In other words, both positive and negative affect can contribute to enhanced creativity motivation in a parallel manner through two different routes. This interpretation is in line with the empirical findings that positive affect is related to approach motivation, while negative affect is associated with avoidance motivation (Wang et al., 2022). In line with this argument, a nonsignificant correlation coefficient between PA and NA was found in this study. These findings revealed that both positive and negative affect are important to creativity motivation, which suggests that optimal creativity performance can result from the interplay between and joint effects of positive and negative affect (see also Baas, 2019; Madrid and Patterson, 2021).

It is interesting that the findings obtained in Studies 1 and 2 consistently demonstrated a greater impact of positive affect than negative affect on creativity motivation. These findings can be interpreted in light of core affect theory (Russell, 2003) and valence-arousal theory (Baas et al., 2008; Sambrano et al., 2021), which conceptualize affect as an integral blend of arousal (sleepy–activated) and hedonic tone (pleasure–displeasure). These theories emphasize that the effect of affect on psychological functioning cannot be understood solely in terms of valence. Rather, arousal (or activation intensity) and valence (or hedonic tone) interact with each other to determine the relevant processes and outcomes. Indeed, an increasing number of empirical studies (e.g., Chi et al., 2021; Frith et al., 2022; Smith et al., 2022) have supported the critical role of activation intensity with respect to moderating the effect of positive and negative valence on creative functioning. It was found that both high-activated positive affect (e.g., happiness) and high-activated negative affect (e.g., anger) are associated with a higher level of creative performance than low-activated positive affect (e.g., relaxation) and low-activated negative affect (e.g., sadness). These findings support the account that the affect-creativity link can be better understood as a function of an affective state’s associated level of activation (Baas, 2019).

In line with this possible account, it is interesting to observe that all five PA items (i.e., active, alert, attentive, determined, and inspired) in the mood instrument used in the present research belong to the category of high-activated positive affect. While a majority of the five NA items (i.e., afraid, ashamed, hostile, nervous) belong to the category of high-activated negative affect, there is also one item (i.e., upset) from the category of deactivating negative affect (see Madrid and Patterson, 2018). With respect to the materials used in emotion induction, the musical stimuli employed for emotion induction were Mozart’s Sonata for Two Pianos in D Major and Albinoni’s Adagio in G Minor, which are known to be linked to emotional feelings of happiness and sadness, respectively (e.g., Fox and Moore, 2021). While happiness is a high-activated positive affect, sadness is a low-activated negative affect (He and Wong, 2022). These observations suggest that in both investigations conducted for this research, only high-activated positive affect was examined regarding its role in creativity motivation. With respect to negative affect, however, the roles of both high-and low-activated affect were examined.

In fact, the findings of this research regarding the changes in arousal scores revealed that the positive emotion induction group showed a stronger effect on increased activation levels than the negative emotion induction group. Regarding the changes in the feeling of pleasure or displeasure, the results also suggest a stronger effect for the positive emotion induction group with respect to the feeling of an increased level of pleasure than for the negative emotion induction group with respect to the feeling of an increased level of displeasure. These results potentially confirm that the samples in this study may experience a higher activation intensity from positive affective states than from negative affective states, which in turn causes the former to have a stronger effect on increased creativity motivation. Further empirical investigation is warranted to test this speculation.

Despite these interesting findings, several limitations of the study should be noted. First, to test moods, the I-PANAS-SF was employed. While this instrument has received a great deal of support regarding its psychometric properties, it measures only some aspects of affect. For instance, all of the PA items measure only high-activated positive affect, and the majority of the NA items measure high-activated negative affect. However, affect is not a unitary phenomenon, and it varies widely in both hedonic tone and activation intensity. Future studies should consider alternative mood measures with the aim of understanding the general relation between affect and creativity motivation. In relation to the use of the I-PANAS-SF to assess the baseline habitual mood in Study 2 to control for its possible covariate effect on creativity motivation, future studies should also consider measuring habitual mood in the posttest condition to control for its possible covariate effect. Second, in the emotion induction procedure, only musical stimuli were used. More specifically, the musical stimuli used in this study were Mozart’s Sonata for Two Pianos in D Major and Albinoni’s Adagio in G Minor, which are linked to emotional feelings of happiness and sadness, respectively. While happiness represents a high-activated positive affect and sadness represents a low-activated negative affect, future research should address whether the findings obtained by the present research can be generalized to other types of affective states, for instance, low-activated positive affect (e.g., relaxation, calmness, comfort) or high-activated negative affect (e.g., anger, irritation, disgust). Moreover, while an increasing number of researchers have highlighted the importance of studying the effect of specific and discrete emotions on creativity (e.g., Baas, 2019; Park et al., 2019), future research should further explore the link between specific and discrete emotions and creativity motivation.

Third, while the present study focused on examining the roles of two types of affective experiences in a newly developed construct (i.e., creativity motivation), it is important that a complete test of a model be conducted with respect to the relationships among the three variables (i.e., affect, creativity motivation, creativity outcomes) to enrich the understanding regarding the direct and indirect roles of affective experiences in creativity outcomes. It would also be interesting to further explore the effect of critical moderating variables (e.g., contextual and personality factors) in modifying the paths in the model. Finally, the fourth limitation concerns the nature of the study samples. While this research involved both an adolescent sample and an emerging adult sample, all participants were Chinese students in Hong Kong. Future studies should extend this line of research to participants with diverse ethnic and socioeconomic backgrounds, which can help test the generalizability of the findings obtained in this study.

## Conclusion

Prior limitations notwithstanding, the present study makes a significant contribution to the literature with respect to the role of affect in creativity motivation. While previous affect-creativity research has focused predominantly on the role of affect in the cognitive components of creativity and yielded mixed results, this research adds to the literature by demonstrating the beneficial role of affect in creativity motivation. By conducting two investigations that involved different affective variables, different methodologies, and across student samples of different age groups ranging from adolescents to emerging adults, empirical evidence was consistently found to support the motivational benefits of both trait-link (i.e., mood) and state-like affects (i.e., emotional state) in both a positive and a negative valence. These findings carry educational implications that students’ creativity motivation can be influenced by a broad spectrum of affective experiences (i.e., positive and negative affect, stable and enduring moods, and momentary and mutable emotions). Encouraging students to accept and appreciate the diversity of affective experiences may be a first step for enhancing their creativity motivation.

## Data availability statement

Requests to access the datasets should be directed to the corresponding author.

## Ethics statement

The studies involving human participants were reviewed and approved by The Human Research Ethics Committee of the Education University of Hong Kong. Written informed consent to participate in this study was provided by the participants and/or the participants’ legal guardian/next of kin.

## Author contributions

W-jH contributed to the conception and design of the work as well as the acquisition, analysis, and interpretation of the data. She contributed to the article and approved the submitted version.

## Funding

The work described in this article was partially supported by a grant from the Education University of Hong Kong (Project No: RG57/2019-2020R).

## Conflict of interest

The author declares that the research was conducted in the absence of any commercial or financial relationships that could be construed as a potential conflict of interest.

## Publisher’s note

All claims expressed in this article are solely those of the authors and do not necessarily represent those of their affiliated organizations, or those of the publisher, the editors and the reviewers. Any product that may be evaluated in this article, or claim that may be made by its manufacturer, is not guaranteed or endorsed by the publisher.
